# The untapped potential of physical activity monitoring for quality assurance of field-based walking tests in clinical respiratory trials

**DOI:** 10.1177/14799731221089318

**Published:** 2022-04-05

**Authors:** Mark W Orme, Ilaria Pina, Sally J Singh

**Affiliations:** 1 Department of Respiratory Sciences, 4488 University of Leicester, Leicester, UK; 2 Centre for Exercise and Rehabilitation Science, University Hospitals of Leicester NHS Trust, NIHR Leicester Biomedical Research Centre-Respiratory, Leicester, UK

**Keywords:** Physical activity, field-based walking tests, incremental shuttle walking test, endurance shuttle walk test, accelerometry, measurement properties, quality assurance, 6-minute walk test

## Abstract

Field-based walking tests are well-established outcome measures in clinical research trials and in the evaluation of routine clinical services, including pulmonary rehabilitation. Despite widespread use, there has been little attention to, or reporting of, the quality assurance of these tests. Physical activity monitoring has become increasingly popular and data from activity monitors could be used for quality assurance of field-based walking tests. We provide examples in this article of data-driven insights possible with this approach, using data from waist-worn accelerometry, for the incremental shuttle walking test (ISWT), endurance shuttle walk test (ESWT) and six-minute walk test (6MWT). Given the multitude of devices to measure physical activity and the range metrics to describe physical activity, we also comment on some of the technical considerations to using activity monitors for walking test quality assurance. Data-driven approaches to quality assurance are already commonplace for other outcome measures in clinical respiratory trials, but little is known about this approach for field-based walking tests. The application of physical activity monitoring may be extended to other field-based exercise tests and additional rehabilitation services. This may be more challenging for self-paced walking tests such as the 6MWT. Future work should apply this approach to research trials and service evaluations to explore the impact of field-based walking test quality on performance (e.g. distance on the ISWT or time achieved for the ESWT), responsiveness to interventions (e.g. pulmonary rehabilitation) and effectiveness of training procedures (e.g. remote training for multi-site trials).

The use of standardised field-based walking tests are fundamental to understanding physiological responses to exercise for people living with chronic respiratory diseases (CRDs).^1^ The most common field-based exercise tests include the incremental shuttle walking test (ISWT),^2^ endurance shuttle walk test (ESWT)^3^ and six-minute walk test (6MWT).^4^ Field-based walking tests are well-established outcome measures in clinical research trials and in the evaluation of routine clinical services, including pulmonary rehabilitation. Despite widespread use, there has been little attention to, or reporting of, the quality assurance of these tests. Physical activity monitoring has become increasingly popular and data from activity monitors could be used for quality assurance of field-based walking tests.

Despite standard operating procedures (SOPs) and technical standards providing quality assurance procedures, there may be other influences that can impact the quality, and therefore performance, of these tests. These may include persistent difficulties of patients timing their walk with the audio signals, poor operator guidance, unintentional impacts of tone/volume of standardised instructions, and external environmental factors such as unexpected corridor traffic or poor lighting. For the ISWT, some patients also have a tendency to walk faster than the required speed, not only in early levels, but even at later stages in spite of guidance from the operator. For the 6MWT, which is a self-paced walking test, quality assurance is less intuitive. However, differences in the pattern of walking during and between tests are quantifiable. Patients’ performance may be influenced by variations in walking speed and rest periods during the 6 minutes. These factors, as well as a broader evaluation of SOP compliance, may be possible to identify through activity monitoring during the tests.

Physical activity monitoring using devices such as accelerometers has become increasingly popular, with the use of PA metrics as clinical trial endpoints continuing to grow in CRD research.^5,6^ Many studies, including large multi-site/multi-national trials, collect field-based walking tests and device-based physical activity, but there is limited data integrating activity monitors within these field-based walking tests.^7^ We propose that data from accelerometers worn during the tests can add further insight.

To illustrate the concept, we provide data during an ISWT, ESWT and 6MWT collected using an ActiGraph wGT3x-BT accelerometer, initialized to collect data at 100 Hz, worn on the waist and processed using vertical axis counts in 10-second epochs. Data were processed using ActiLife 6 software (ActiGraph Inc, Pensacola, FL, USA) and Microsoft Excel 365. Figure 1 provides four scenarios captured by PA monitoring. Panel A (two ISWTs and one ESWT conducted on the same day) shows an example of gold-standard testing, including adequate rest periods and adherence of the patient to the walking speed of the respective levels. In Panel B, it is possible to identify a patient who has found it challenging to walk in time with the required speeds of the ISWT, including setting off too quickly at the beginning. In Panel C, a disruption to the ESWT can be seen which has caused the patient to come to a stop. In Panel D, variations in the speed of walking during the 6MWT, including slowing down, resting and increasing their speed, can be detected using the activity monitor. The extent to which these field-based walking tests are performed in accordance with their SOPs are not commonly reported and it therefore remains unclear whether these deviations have an impact on performance or the evaluation of interventions.

**Figure 1.**
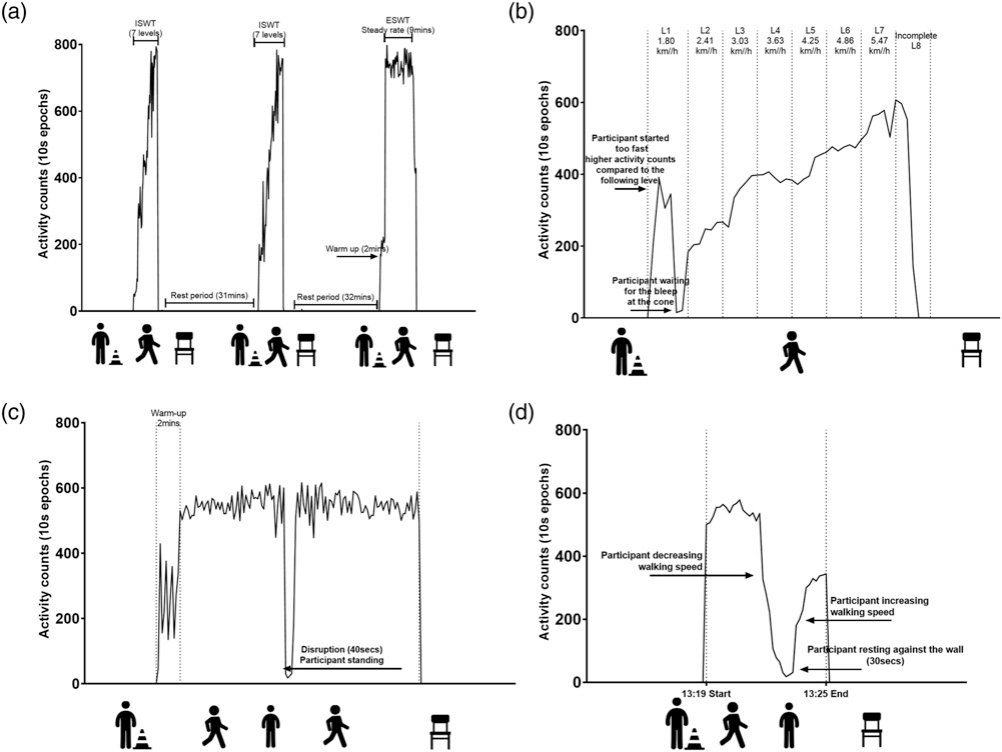
Four walking test scenarios captured by physical activity monitoring. Panel A: Two ISWTs and one ESWT conducted on the same day to demonstrate gold-standard testing, including adequate rest periods and adherence to the walking speeds of the respective levels. Panel B: Identifying a test in which the patient found it challenging to walk in time with the required speeds of the ISWT, including setting off too quickly at the beginning. Panel C: A disruption to the ESWT which caused the patient to come to a stop during the test. Panel D: Variations of walking speed and rest periods during the 6MWT.

There are a multitude of physical activity devices available to clinicians or researchers (e.g. research-grade and commercial devices), with a wide range of wear location (e.g. waist, wrist, upper arm) and a plethora of metrics that can be obtained from the data (e.g. step count, device counts, raw acceleration).^8^ The decision of how to quantify physical activity should therefore be based on the research question. In the context of quality assurance during field-based walking tests, one could argue that waist-worn accelerometers, able to capture acceleration at the centre of mass and at high resolution may be best suited. Higher resolution data will be more sensitive to subtle changes in walking intensity that are missed with longer epoch lengths.^9^ In practice, with sufficient resolution, the use of step count (cadence) or acceleration will allow meaningful quality assessments. Physiological parameters such as metabolic equivalent of task or heart rate will not be sensitive enough to detect variations in these tests. For example, they do not have a ‘true zero’ to detect absences of movement such as rest periods during the 6MWT or waiting at the cones during the ISWT or ESWT. With the wide adoption of our proposed approach, it will become possible to develop a quality assurance framework for field-based walking tests using activity monitors.

The concept of such an independent, data-driven approach to data quality is not a new one. Validation ranges for blood tests and automated quality checks during spirometry procedures are commonplace. As with all measures of this type, there is inevitable variability within and between operators, which may be important to account for in the evaluation of treatments or interventions. It is good practice for study team members to conduct measurements alongside expert examiners to reinforce SOPs (e.g. single-site or multi-site measurement competency workshops). Inter- and intra-observer errors may be calculated, reported and used as part of analytical processes.^10^ There may be further value of adopting a similar approach for field-based walking tests for multi-site/multi-national trials, with variations in the environments in which the tests are conducted and variations in prior operator experience potentially influencing participant performance. The integration of physical activity monitoring within field-based walking tests can be during research visits or routine clinical appointments (e.g. pulmonary rehabilitation assessments).

Future trials and service evaluations should integrate physical activity monitoring within the conduct of field-based walking tests to explore the impact of test quality on performance, responsiveness to interventions and effectiveness of training procedures.

## Notation

CRD: Chronic respiratory disease
ESWT: Endurance shuttle walk test
ISWT: Incremental shuttle walking test
PA: Physical activity
PR: Pulmonary rehabilitation
SOP: Standard operating procedure
Technical note
